# *In Vitro* Acid-Mediated Initial Dental Enamel Loss Is Associated with Genetic Variants Previously Linked to Caries Experience

**DOI:** 10.3389/fphys.2017.00104

**Published:** 2017-02-22

**Authors:** Alexandre R. Vieira, Merve Bayram, Figen Seymen, Regina C. Sencak, Frank Lippert, Adriana Modesto

**Affiliations:** ^1^Department of Oral Biology, School of Dental Medicine, University of PittsburghPittsburgh, PA, USA; ^2^Department of Pediatric Dentistry, School of Dental Medicine, University of PittsburghPittsburgh, PA, USA; ^3^Department of Pedodontics, School of Dentistry, Medipol Istanbul UniversityIstanbul, Turkey; ^4^Department of Pedodontics, School of Dentistry, Istanbul UniversityIstanbul, Turkey; ^5^Department of Cariology, Operative Dentistry and Dental Public Health, School of Dentistry, Indiana UniversityIndianapolis, IN, USA

**Keywords:** dental caries, dental enamel, dental erosion, transcription factors, aquaporins

## Abstract

We have previously shown that *AQP5* and *BTF3* genetic variation and expression in whole saliva are associated with caries experience suggesting that these genes may have a functional role in protecting against caries. To further explore these results, we tested *ex vivo* if variants in these genes are associated with subclinical dental enamel mineral loss. DNA and enamel samples were obtained from 53 individuals. Enamel samples were analyzed for Knoop hardness of sound enamel, integrated mineral loss after subclinical carious lesion creation, and change in integrated mineral loss after remineralization. DNA samples were genotyped for single nucleotide polymorphisms using TaqMan chemistry. Chi-square and Fisher's exact tests were used to compare individuals above and below the mean sound enamel microhardness of the cohort with alpha of 0.05. The A allele of *BTF3* rs6862039 appears to be associated with harder enamel at baseline (*p* = 0.09), enamel more resistant to demineralization (*p* = 0.01), and enamel that more efficiently regain mineral and remineralize (*p* = 0.04). Similarly, the G allele of *AQP5* marker rs3759129 and A allele of *AQP5* marker rs296763 are associated with enamel more resistant to demineralization (*p* = 0.03 and 0.05, respectively). *AQP5* and *BTF3* genetic variations influence the initial subclinical stages of caries lesion formation in the subsurface of enamel.

## Introduction

Dental caries is a complex multifactorial disease and historically the biological factors operating within the host have been less explored. Our group has, for the last decade, investigated the possible role of genetic variation in the individual susceptibility to caries. Our results suggest, as expected, that several genes underlying multiple mechanisms (i.e., enamel formation, immune response, saliva composition, and quantity) are associated with dental caries (reviewed in Nibali et al., 2016).

One methodological challenge has been the use of past and current caries experience as a measure of disease. We believe that caries experience indicators do not capture fully the underlying mechanisms modulating the pathogenesis of dental caries in an individual, which may hinder discovery if one is trying to identify factors increasing or decreasing individual susceptibility to disease. With that idea in mind, we started to define the disease based on subclinical enamel loss (Shimizu et al., 2012; Weber et al., 2014; Bayram et al., 2015; Vieira et al., 2015) or presence of periapical pathology related to deep caries lesions in dentin (Menezes-Silva et al., 2012; Dill et al., 2015; Maheshwari et al., 2016). Typically, sound enamel microhardness is a poor indicator for susceptibility to demineralization (Lippert and Lynch, 2014). A myriad of factors are involved in the caries process and focusing only on enamel, structural (e.g., pore size and volume, ratio between interprismatic and prismatic enamel fractions) and compositional differences (e.g., Mg, Na, CO_3_, F contents) are likely the predetermining factors for caries susceptibility. We studied sound enamel microhardness presently to gain further insight into potential genetic factors predetermining susceptibility to demineralization and to explain variations in sound enamel microhardness. Here we are expanding this work to two loci that we previously showed are associated with caries experience, 5q13.2 and 12q13.12, to verify if we can still detect associations when a different phenotypical definition for dental caries is used.

## Methods

This study was approved by the Ethics Committee of the Istanbul University, Medical Faculty, Istanbul, Turkey and the University of Pittsburgh Institutional Review Board (IRB# 11070236). Written informed consent was obtained from all participating individuals and parents/legal guardians. Fifty-three orthodontic patients from Istanbul University, Faculty of Dentistry, Department of Orthodontics, participated in this study during the period 5 September 2011 to 30 November 2012. Participants had an indication for extraction of pre-molars for orthodontic reasons and were consecutively invited to participate in the study during the period described above. They agreed to donate their extracted tooth (teeth). One first premolar, extracted for orthodontic reasons, was used from each participant as a source of enamel.

Unstimulated saliva samples were obtained from all participants and stored in Oragene DNA Self-Collection kits (DNA Genotek, ON, Canada) at room temperature until processed. DNA was extracted according to the manufacturer's instructions. Ten single nucleotide polymorphisms (SNPs) were selected, including six in the aquaporin locus 12q13.2 (rs3759129, rs10875989, rs1996315, rs2878771, rs296763, and rs467323) and four located in 5q13.2 (rs27565, rs4700418, rs875459, and rs6862039). These SNPs were chosen based on the results of our previous fine mapping studies on the two loci (Shimizu et al., 2013; Anjomshoaa et al., 2015). Polymerase chain reactions with TaqMan (hydrolysis probes that are designed to increase the specificity of quantitative PCR) SNP Genotyping Assays from Applied Biosystems (Valencia, CA, USA), with a total volume of 3 μl per reaction and 3.0 ng of DNA per reaction, were used for genotyping all selected markers in a Tetrad PTC225 thermocycler from MJ Research (Waltham, MA, USA). Genotype detection and analysis were performed using the ABI 7900HT with ABI SDS software (Applied Biosystems, Valencia, CA, USA).

**Figure 1:**
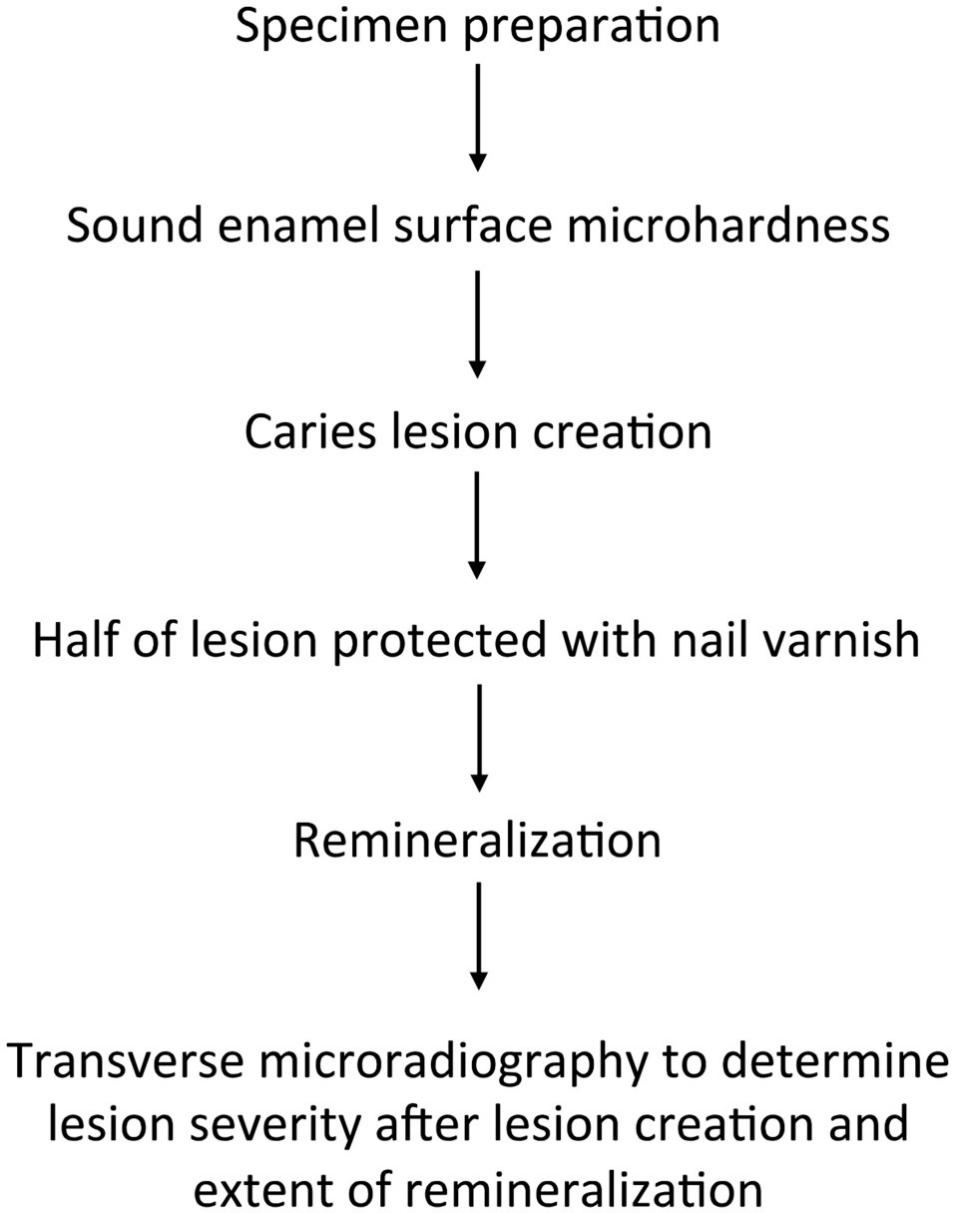
**Summary of the study design**.

Fifty-three caries-free premolar teeth (one from each participant), extracted for orthodontic reasons, were studied. Figure 1 summarizes the study design. Caries experience of this cohort was high, with a mean DMFT score of 5.19.

The tissue remnants were cleaned from the teeth and then teeth were stored in 10% buffered formalin (pH 7.0) solution at 4°C until required for initial laboratory manipulation. The crowns were separated from the roots, and then each tooth was cut into 3 × 3-mm specimens using a low-speed saw (Isomet, Buehler, Lake Bluff, IL, USA). The teeth were stored in thymol during the sample preparation process. The specimens were embedded individually in acrylic resin (Varidur, Buehler) and polished to create flat surfaces to facilitate surface microhardness testing using Struers Rotopol 31/Rotoforce 4 polishing unit (Struers Inc., Cleveland, PA, USA). Specimens were ground flat and polished with water-cooled abrasive disks (500-, 1,200-, 2,400-, and 4,000-grit SiC papers; MDFuga, Struers Inc., Cleveland, Ohio, USA) and polishing cloth with diamond suspension (1 μm; Struers Inc.). After the polishing procedures, specimens were sonicated in neutral detergent solution and rinsed with deionized water. As a final cleaning step, the specimens were sonicated in a detergent solution (Micro-90 concentrated cleaning solution with 2 % dilution) for 3 min. The specimens were finally assessed under Nikon SMZ 1500 stereomicroscope at × 10 magnification. Accepted specimens had no obvious cracks, areas of hypomineralization, or other flaws in the enamel surface.

Initial hardness of each specimen was determined using a Knoop microhardness indenter (2100 HT; Wilson Instruments, Norwood, MA, USA) at a load of 50 g for 15 s. The average specimen surface microhardness was determined from five indentations placed in the center of the surface of each specimen, ~100 μm apart from one another.

Early carious lesions were created in the specimens utilizing a demineralization protocol based on that by White (1987), which has been extensively studied using a variety of techniques over the years (White, 1987; Churchley et al., 2011). Artificial lesions were formed in the enamel specimens of each disk by a 5-day immersion into a solution containing 0.1 M lactic acid, 4.1 mM CaCl_2_ × 2 H_2_O, 8.0 mM KH_2_PO_4_, and 0.2% w/v Carbopol 907 (BF Goodrich Co., USA), pH adjusted to 5.0 using KOH, for 5 days at 37°C. Demineralization was performed at a ratio of 10 ml of solution per specimen. The resulting lesions were early, shallow, subsurface^1^ lesions with a typical, average depth of approximately 50 μm. After lesion creation, approx. half of the lesion surface area was covered with colored, acid-resistant nail varnish to preserve a baseline lesion for future analysis.

All lesions were then remineralized for 4 days at 37°C using “resting plaque fluid” (Lynch et al., 2007) with the following composition: 10 mM acetic acid, 1.0 mM CaCl_2_ × 2 H_2_O, 12.7 mM KH_2_PO_4_, 130 mM KCl, 20 mM HEPES, 0.1 ppm F (NaF), pH adjusted to 6.5 using KOH.

Sections, approximately 100 μm in thickness and two per specimen, were cut from the center of the specimens across the varnish-covered lesion area and remineralized lesion window using a Silverstone-Taylor Hard Tissue Microtome (Scientific Fabrications Laboratories, USA). The sections were mounted, with an aluminum step wedge, on high resolution glass plate Type I A (Microchrome Technology Inc., San Jose, CA) and X-rayed at 20 kV and 30 mA at a distance of 42 cm for 65 min. The film was developed in Kodak D-19 developer for 3 min, placed in a stop bath (Kodak 146-4247) for 45 s, and then fixed (Kodak 146-4106) for 3 min. All plates were then rinsed in deionized water for 15 min and air-dried. Microradiographs were examined with a Zeiss EOM microscope in conjunction with the TMR software v.3.0.0.11. Sound enamel was assumed to be 87% v/v mineral. Integrated mineral loss (ΔZ) was recorded for both the varnish-covered lesion baseline area and the remineralized lesion. The difference was calculated (pre–post data) to assess the extent of remineralization.

Based on sound enamel hardness values, subjects were classified into dichotomous groups (baseline values or rate changes above or below the average of the group). Subjects were classified as having “softer enamel” (below the average of the group) and “harder enamel” (above the average of the group) for determination of hardness phenotypes. Chi-square and Fisher's exact tests were used to assess association between the SNPs and hardness values by the use of the PLINK software package (Purcell et al., 2007) with an established alpha of 0.05.

## Results

Table 1 shows the mean values of hardness and Table 2 summarizes all genotyping frequencies and comparisons were made between individuals that showed levels of enamel loss above or below the mean loss of the studied sample. The A allele of *BTF3* rs6862039 appears to be associated with harder enamel at baseline (*p* = 0.09), enamel more resistant to demineralization (*p* = 0.01), and enamel that more efficiently regain mineral and remineralize (*p* = 0.04). Similarly, the G allele of *AQP5* marker rs3759129 and A allele of *AQP5* marker rs296763 are associated with enamel more resistant to demineralization (*p* = 0.03 and 0.05, respectively). No other markers studied showed statistical evidence of association.

**Table 1:** **Mean hardness of studied specimens**.

**Study phase**	**Baseline**	**After creation of artificial lesion**	**After remineralization**
Mean Enamel Hardness	45.95	64.92	76.62

**Table 2:** **Genotyping frequencies and summary results of the association studies**.

**Locus**	**Marker (rs^#^) and gene**	**Genotype/allele**	**Number of Individuals (Genotypes) or Alleleswith less enamel loss**	**Number of Individuals (Genotypes) or Alleleswith more enamel loss**	***p*****-value**	**Number of Individuals (Genotypes) or Alleleswith less enamel loss**	**Number of Individuals (Genotypes) or Alleleswith more enamel loss**	***p*****-value**	**Number of Individuals (Genotypes) or Alleleswith less enamel loss**	**Number of Individuals (Genotypes) or Alleleswith more enamel loss**	***p*****-value**
			**Baseline**			**After creation of artificial lesion**			**After remineralization**		
5q13.2	27565 (*PART1*)	CC	3	4	0.68^#^	2	6	0.77^#^	5	5	0.89^#^
		CT	13	14		11	19		16	18	
		TT	1	3		1	3		4	3	
		C	19	22	0.76^##^	15	31	0.88^##^	26	28	0.85^##^
		T	15	20		13	25		24	24	
	4700418 (*ZSWIM6*)	CC	5	7	0.71^#^	3	9	0.64^#^	7	7	0.98^#^
		CG	7	6		7	10		11	11	
		GG	5	8		4	9		7	8	
		C	17	20	0.84^##^	13	28	0.76^##^	25	25	0.85^##^
		G	17	22		15	28		25	27	
	875459 (*CCNB1*)	GG	5	5	0.69^#^	2	8	0.3^#^	5	0	0.32^#^
		GT	11	13		9	18		18	2	
		TT	1	3		3	2		2	17	
		G	21	23	0.84^##^	13	34	0.21^##^	28	26	0.54^##^
		T	13	19		15	22		22	26	
	6862039 (*BTF3*)	AA	1	0	0.22^#^	1	0	0.1^#^	2	0	0.32^#^
		AT	2	1		2	1		4	2	
		TT	8	17		7	22		17	17	
		A	4	1	0.04^##^	4	1	0.01^##^	8	2	0.09^##^
		T	18	35		16	45		38	36	
Aquaporin	467323 (*AQP2*)	AA	2	3	0.88#	1	4	0.5^#^	1	4	0.32^#^
12q13.12		AG	8	11		6	15		12	13	
		GG	7	7		7	9		12	9	
		A	12	17	0.64^##^	8	23	0.26^##^	14	21	0.19^##^
		G	22	25		20	33		36	31	
	2878771 (*AQP2*)	AA	2	0	0.26^#^	1	1	0.83^#^	2	0	0.3^#^
		AG	5	8		5	9		7	7	
		GG	10	13		8	18		15	19	
		A	9	8	0.44^##^	7	11	0.57^##^	11	7	0.22^##^
		G	25	34		21	45		37	45	
	10875989 (*AQP2*)	AA	2	3	0.9^#^	1	4	0.26^#^	1	4	0.18^#^
		AG	9	12		6	17		12	15	
		GG	6	6		7	7		12	7	
		A	13	18	0.68^##^	8	25	0.16^##^	14	23	0.09^##^
		G	21	24		20	31		36	29	
	3759129 (*AQP5*)	AA	8	13	0.36^#^	5	19	0.07^#^	14	14	0.91^#^
		AG	6	5		6	6		6	8	
		GG	1	0		1	0		1	1	
		A	22	31	0.19^##^	16	44	0.03^##^	34	36	0.75^##^
		G	8	5		8	6		8	10	
	296763 (*AQP5*)	AA	2	0	0.25^#^	1	1	0.12#	0	2	0.21^#^
		AG	6	7		7	6		11	7	
		GG	9	14		6	21		14	17	
		A	10	7	0.18^##^	9	8	0.05^##^	11	11	1.0^#^
		G	24	35		19	48		39	41	
	1996315 (*AQP6*)	AA	0	0	0.52^#^	0	0	0.93^#^	0	0	0.23^#^
		AG	3	2		3	5		3	6	
		GG	12	15		11	17		20	16	
		A	3	2	0.54^##^	3	5	0.93^##^	3	6	0.26^##^
		G	27	32		25	39		43	38	

# *p-value of the comparison of genotyping frequencies between individuals with enamel loss below and above the mean of the studied group. Chi-square or Fisher's exact tests with 2 degrees of freedom*.

## *p-value of the comparison of allele frequencies between individuals with enamel loss below and above the mean of the studied group. Chi-square or Fisher's exact tests with 1 degree of freedom*.

## Discussion

Our data confirm the association between dental caries and genetic variation in *BTF3* and *AQP5* we have previously reported (Shimizu et al., 2013; Anjomshoaa et al., 2015). When we reported the association between caries experience and *BTF3* (Shimizu et al., 2013), we tested if *BTF3* was expressed in whole saliva and if expression that correlated with caries experience. We suggested based on our initial findings that BTF3 may be involved in caries susceptibility acting through saliva. This gene encodes the basic transcription factor 3, a protein that forms a stable complex with RNA polymerase IIB and is required for transcriptional initiation. Alternative splicing results in multiple transcript variants encoding different isoforms. *BTF3* has multiple pseudogenes (https://www.ncbi.nlm.nih.gov/gene, Gene ID = 689). In gastric cancer, BTF3 expression is associated with enhanced cell proliferation, reduced cell cycle regulation, and apoptosis and its silencing inhibits proliferation of gastric cancer cells (Liu et al., 2013). Our data may suggest that *BTF3* may be involved in the formation of the enamel, possibly acting as a transcription factor controlling cell proliferation.

Aquaporin 5 (AQP5) is a water channel protein expressed in salivary and lacrimal glands, various types of epithelial cells, and during tooth development (Ishida et al., 1997; Nielsen et al., 1997; Funaki et al., 1998; Hamann et al., 1998; Felszeghy et al., 2004). AQP5 interactions during dental development may impact the formation of dental enamel and susceptibility to dental caries (Anjomshoaa et al., 2015). Since the AQP locus was not associated with enamel hardness, we thought the role of AQP5 in caries was through salivation rather than influencing enamel development. We decided to reassess our microhardness experiments and this time we tested the specimens, instead directly at the treated surface, in the subsurface. These analyses suggested that genetic variants in AQP5 are associated with initial enamel loss, which is a surrogate for the development of early caries lesions. These data support the idea that *AQP5* impacts enamel development possibly making it more susceptible to caries.

While concerned about multiple testing, we avoided to apply the strict Bonferroni correction and increase type II error. If we had used Bonferroni correction, we would have lowered the alpha to 0.005 (0.05/10). We have demonstrated previously (Vieira et al., 2008) that known true associations are missed when correction for multiple testing is implemented. The results of our work should be considered with caution and serve to generate a hypothesis to be directly tested in larger and more homogeneous samples. On the other hand, simply disregarding the nominal associations presented here may delay discovery by misleading the field to believe that no true biological relationships exist. Another limitation of our study is that our phenotype reflects subclinical caries lesions that cannot be detected by the naked eye (Shimizu et al., 2012). The SNPs we report here as associated with caries do not have known functional roles. Our work continues to support that individuals susceptibility is a factors in dental caries susceptibility, with some individuals more susceptible to mineral losses when pH lowers.

## Author contributions

ARV designed the study, obtained support, analyzed and interpreted data, and wrote the first draft of the manuscript. MB and FS (Istanbul University) helped design the study facilitated DNA and sample collections, Kathleen Deeley (University of Pittsburgh) managed samples and generated genotypes, FL (Indiana University) helped design the study and generated enamel microhardness measurements, and RS (University of Pittsburgh) statistically analyzed the data. AM helped design the study and obtained support. All authors critical revised the manuscript.

### Conflict of interest statement

The authors declare that the research was conducted in the absence of any commercial or financial relationships that could be construed as a potential conflict of interest. The reviewer CC and handling Editor declared their shared affiliation, and the handling Editor states that the process nevertheless met the standards of a fair and objective review.

## Footnotes

^1^The chosen demineralization procedure will result in the formation of early caries lesions. The most prominent feature of these lesions is that demineralization is primarily confined to enamel mineral below the surface. Hence, the term “subsurface” demineralization has been introduced into the literature to differentiate to surface loss such as that occurring during dental erosion.
